# Convergence in $s_{2}$-quasicontinuous posets

**DOI:** 10.1186/s40064-016-1873-6

**Published:** 2016-02-29

**Authors:** Xiao-jun Ruan, Xiao-quan Xu

**Affiliations:** Department of Mathematics, Sichuan University, Chengdu, 610064 Sichuan People’s Republic of China; Department of Mathematics, Nanchang University, Nanchang, 330031 Jiangxi People’s Republic of China; College of Mathematics and Information Science, Jiangxi Normal University, Nanchang, 330022 Jiangxi People’s Republic of China

**Keywords:** $s_2$-Continuous poset, $s_2$-Quasicontinuous poset, Weak Scott topology, ${\mathcal {S}}$-Convergence, ${\mathcal {GS}}$-Convergence, 06B35, 06B75, 54F05

## Abstract

In this paper, we present one way to generalize ${\mathcal {S}}$-convergence and ${\mathcal {GS}}$-convergence of nets for arbitrary posets by use of the cut operator instead of joins. Some convergence theoretical characterizations of $s_2$-continuity and $s_2$-quasicontinuity of posets are given. The main results are: (1) a poset *P* is $s_2$-continuous if and only if the ${\mathcal {S}}$-convergence in *P* is topological; (2) *P* is $s_2$-quasicontinuous if and only if the ${\mathcal {GS}}$-convergence in *P* is topological.

## Background

The theory of continuous domains, due to its strong background in computer science, general topology and logic has been extensively studied by people from various areas (see Abramsky and Jung 1994; Gierz et al. 1980, 2003). Since many models may not be dcpos, an important direction in the study of continuous domains is to extend the theory of continuous domains to that of posets as much as possible (see Huang et al. 2009; Lawson and Xu 2004; Mislove 1999; Markowsky 1981; Mao and Xu 2006; Venugopalan 1990; Zhang 1993; Zhang and Xu 2015). It has turned out to be very fruitful for many categorical and topological developments generalizing the theory of continuous domains, but it is still rather restrictive, taking into consideration only the case of existing a join. Furthermore, it fails to be completion-invariant, that is, the normal completion of a continuous poset is not always a continuous lattice, which means some useful information of subsets whose joins do not exist has been thrown away in some sense. In 1981, Erné introduced the concept of $s_2$-continuous posets in terms of the cut operator instead of joins. The notion of $s_2$-continuity admits to generalize most important characterizations of continuity from dcpos to arbitrary posets and has the advantage that not even the existence of directed joins has to be required. As a generalization of $s_2$-continuity, the concept of $s_2$-quasicontinuity was introduced by Zhang and Xu (2015), their basic idea is to generalize the way below relation between the points to the case of sets. It was proved that $s_2$-quasicontinuous posets equipped with the weak Scott topologies are precisely the hypercontinuous lattices.

Various kinds of convergent classes in posets were studied in Gierz et al. (2003), Zhao and Zhao (2005), Zhou and Zhao (2007), Wang and Zhao (2013), Zhao and Li (2006), Zhou and Li (2013), Chen and Kou (2014). By different convergence, not only are many notions of continuity characterized, but also they make order and topology across each other. In Gierz et al. (2003), the concept of ${\mathcal {S}}$-convergence for dcpos was introduced by Scott to characterize continuous domains. It was proved that for a dcpo, the ${\mathcal {S}}$-convergence is topological if and only if it is a continuous domain. In this paper, making a slight modification of ${\mathcal {S}}$-convergence, we generalize the concept of ${\mathcal {S}}$-convergence to the setting of arbitrary poset by means of the cut operator instead of joins. It is proved that the ${\mathcal {S}}$-convergence in a poset is topological if and only if the poset is $s_2$-continuous. Although Erné investigated the ${\mathcal {S}}$-convergence through filter, we would give a satisfactory sufficient and necessary condition for the ${\mathcal {S}}$-convergence to be topological by the net, which is more simple and direct than the filter. In order to characterize the $s_2$-quasicontinuity we shall also consider another type of ${\mathcal {GS}}$-convergence in a poset, and get the desired result that the ${\mathcal {GS}}$-convergence in a poset is topological if and only if the poset is $s_2$-quasicontinuous.

## Preliminaries 

Let *P* be a partially ordered set (poset, for short). We put $P^{(<\omega )}=\{F\subseteq P : F\ \text{ is } \text{ finite }\}$. For all $x\in P$, $A\subseteq P$, define $\downarrow x=\{y\in P: y\le x\}$ and $\downarrow A=\{x\in P: x\le a$ for some $a\in A\}$; $\uparrow x$ and $\uparrow A$ are defined dually. $A^\uparrow$ and $A^\downarrow$ denote the sets of all upper and lower bounds of *A*, respectively. A cut operator $\delta$ is defined by $A^{\delta } =(A^\uparrow )^\downarrow$ for every $A\subseteq P$. Notice that whenever *A* has a join (supremum) then $x \in A^{\delta }$ means $x\le \vee A$.

For a poset *P*, a subset *U* of *P* is called Scott open if (i) $U=\uparrow U$, and (ii) if *D* is a directed set of *P* and $\vee D\in U$ whenever $\vee D$ exists, then there is some $d\in D$ with $d\in U$. It is easy to see that all the Scott open subsets of *P* form a topology, which we shall call the *Scotttopology*, denoted by $\sigma (P)$.

Let *P* be a poset. We order the collection of nonempty subsets of *P* by $G\le H$ if $\uparrow H\subseteq \uparrow G$. We say that a nonempty family of sets is *directed* if given $F_1$, $F_2$ in the family, there exists *F* in the family such that $F_1$, $F_2\le F$, i.e., $F\subseteq \uparrow F_1\cap \uparrow F_2$. For nonempty subsets *F* and *G* of *P*, we say *FapproximatesG* if for every directed subset $D\subseteq P$, whenever $\vee D$ exists, $\vee D\in \uparrow G$ implies $d\in \uparrow F$ for some $d\in D$. A dcpo *P* is called a *quasicontinuousdomain* if for all $x\in P$, $\uparrow x$ is the directed (with respect to reverse inclusion) intersection of sets of the form $\uparrow F$, where *F* approximates $\{x\}$ and *F* is finite. In particular, a poset *P* is called a continuous poset if for all $x\in P, x$ is the directed supremum of sets of the form *y*, where $\{y\}$ approximates $\{x\}$.

### **Definition 1**

(Erné 1981) Let *P* be a poset.

1. For any $x, y\in P$, we say that *x* is way below *y*, written $x\ll y$ if for all directed sets $D\subseteq P$ with $y\in D^\delta$, there exists $d\in D$ such that $x\le d$. The set $\{y\in P:y\ll x\}$ will be denoted by $\Downarrow x$ and $\{y\in P:x\ll y\}$ denoted by $\Uparrow x$.
2. *P* is called $s_2$-continuous if for all $x\in P$, $x\in (\Downarrow x)^\delta$ and $\Downarrow x$ is directed.

Indeed, we have $x=\vee \Downarrow x$ iff $x\in (\Downarrow x)^\delta$ by $\Downarrow x\subseteq \downarrow x$.

Let us note that an $s_2$-continuous poset is continuous, but the converse may not be true:

### *Example 1*

(*Example 1.7 (1) in* Erné 1981) Consider the Euclidean plane ${\mathbb {R}}\times {\mathbb {R}}$ under the usual order. It is a continuous poset, but it is not $s_2$-continuous, because every lower half-plane

$$\begin{aligned} E_{a}=\{(x, y)\in {\mathbb {R}}\times {\mathbb {R}}: y\le a\} \end{aligned}$$

is a directed lower set with $E_{a}^{\delta }={\mathbb {R}}\times {\mathbb {R}}$, while $\bigcap \{E_{a}: a\in {\mathbb {R}}\}=\emptyset$, hence $\ll$ is empty.

The following lemma shows that the $s_2$-continuous poset has the interpolation property.

### **Lemma 1**

(Erné 1981) *LetPbe an*$s_2$-*continuous poset and*$x, y\in P$. *If*$x\ll y$, *then there is some*$z\in P$*such that*$x\ll z\ll y$.

### **Definition 2**

(Erné 1981, 2009) Let *P* be a poset. A subset $U\subseteq P$ is called weak Scott open if it satisfies

1. $$U=\uparrow U$$;
2. For all directed sets $D\subseteq P$, $D^\delta \cap U\ne \emptyset$ implies $D\cap U\ne \emptyset$.

The collection of all weak Scott open subsets of *P* forms a topology, it will be called the weak Scott topology of *P* and will be denoted by $\sigma _2(P)$.

### *Remark 1*

$\sigma _2(P)$ is always coarser than $\sigma (P)$, and both topologies coincide on dcpos.

### *Example 2*

(*Example 2.5 in *Erné 1981) Consider three disjoint countable sets $A=\{a_{n}: n\in \mathbf {N_{0}}\}, B=\{b_{n}: n\in \mathbf {N_{0}}\}, C=\{c_{n}: n\in {\mathbf {N}}\}$, and the order $\le$ on $P=A\cup B\cup C$ is defined as follows:

- $$\downarrow a_{0}=\{a_{0}\}\cup B$$,
- $$\downarrow a_{n}=\{b_{m}: m<n\}(n\in {\mathbf {N}}, n\ne 2)$$,
- $$\downarrow a_{2}=\{b_{0}, b_{1}\}\cup C$$,
- $$\downarrow b_{n}=\{b_{n}\}(n\in \mathbf {N_{0}})$$,
- $$\downarrow c_{n}=\{c_{m}: m\le n\}(n\in {\mathbf {N}})$$,
- $$x\le y\Leftrightarrow x\in \downarrow y$$.

Then $\uparrow b_{0}$ is open in $\sigma (P)$ but not in $\sigma _2(P)$ since $C=\{c_{n}: n\in {\mathbf {N}}\}$ is a directed lower set with $b_{0}\in C^{\delta }\cap \uparrow b_{0}\ne \emptyset$ while $C\cap \uparrow b_{0}=\emptyset$. Hence in this example, we have $\sigma _2(P)$ is proper contained in $\sigma (P)$.

### **Definition 3**

(Zhang and Xu 2015) Let *P* be a poset and *G*, $H\subseteq P$, we say that *G* is way below *H* and write $G\ll H$ if for all directed sets $D\subseteq P$, $\uparrow H\cap D^\delta \ne \emptyset$ implies $\uparrow G\cap D\ne \emptyset$. We write $G\ll x$ for $G\ll \{x\}$ and $y\ll H$ for $\{y\}\ll H$. The set $\{x\in P:F\ll x\}$ will be denoted $\Uparrow F$.

### **Definition 4**

(Zhang and Xu 2015) Let *P* be a poset. *P* is called $s_2$-*quasicontinuous* if for each $x\in P$, $w(x)=\{F\subseteq P:F\in P^{(<\omega )}$ and $F\ll x\}$ is directed and $\uparrow x=\bigcap \{\uparrow F:F\in w(x)$ }.

Obviously, the $s_2$-continuous is $s_2$-quasicontinuous, but the converse may not be true.

### *Example 3*

(Zhang and Xu 2015) Let $P=\{a\}\cup \{a_n:n\in {\mathbf {N}}\}$. The partial order on *P* is defined by setting $a_n<a_{n+1}$ for all $n\in {\mathbf {N}}$, and $a_1< a$. Then *P* is an $s_2$-quasicontinuous poset which is not $s_2$-continuous.

The following theorem shows that the $s_2$-quasicontinuous poset has the interpolation property.

### **Theorem 1**

(Zhang and Xu 2015) *LetPbe an*$s_2$-*quasicontinuous poset and*$K\in P^{(<\omega )}$, $H\subseteq P$. *If*$H\ll K$, *then there exists a finite setFsuch that*$H\ll F\ll K$.

### **Lemma 2**

(Zhang and Xu 2015) *Let*${\mathcal{F}}$*be a directed family of nonempty finite sets in a poset*. *If*$G\ll x$*and*$\bigcap \nolimits _{F\in {\mathcal {F}}}\uparrow F\subseteq \uparrow x$, *then*$F\subseteq \uparrow G$*for some*$F\in {\mathcal {F}}$.

### **Lemma 3**

(Zhang and Xu 2015) *LetPbe an*$s_2$-*quasicontinuous poset*.

1. *For any nonempty setHinP*, $\Uparrow H=int_{\sigma _2(P)}\uparrow H$.
2. *A subsetUofPis weak Scott open iff for each*$x\in U$*there exists a finite*$F\ll x$*such that*$\uparrow F\subseteq U$. *The sets*$\{\Uparrow F:F\in P^{(<\omega )}\}$*form a basis for the weak Scott topology*$\sigma _2(P)$.

The following lemma is well-known Rudin Lemma.

### **Lemma 4**

(Gierz et al. 2003) *Let*${\mathcal{F}}$*be a directed family of nonempty finite subsets of a posetP*. *Then there exists a directed set*$D\subseteq \bigcup \nolimits _{F\in {\mathcal {F}}}F$*such that*$D\cap F\ne \emptyset$*for all*$F\in {\mathcal {F}}$.

## ${\mathcal {S}}$-Convergence in $s_2$-continuous posets 

In this section, the concept of ${\mathcal {S}}$-convergence in a poset is introduced. It is proved that the poset *P* is $s_2$-continuous if and only if the ${\mathcal {S}}$-convergence in *P* is topological.

### **Definition 5**

Let *P* be a poset and $(x_{j})_{j\in J}$ a net in *P*.

1. A point $y\in P$ is called an eventual lower bound of a net $(x_{j})_{j\in J}$ in *P*, if there exists $k\in J$ such that $y\le x_{j_{}}$ for all $j\ge k$;
2. A point $$x\in P$$ is called an $${\mathcal {S}}$$-limit of the net $$(x_{j})_{j\in J}$$ if there exists some directed set *D* of eventual lower bounds of a net $$(x_{j})_{j\in J}$$ such that $$x\in D^{\delta }$$. We also say $$(x_{j})_{j\in J}$$$${\mathcal {S}}$$ converges to *x* and write $$x\equiv _{{\mathcal {S}}}$$lim $$x_{j}$$.

Let ${\mathcal {S}}$ denote the class of those pairs $((x_{j})_{j\in J}, x)$ with $x\equiv _{{\mathcal {S}}}$lim $x_{j}$, then $\mathcal {O({\mathcal {S}})}=\{U\subseteq P:$ whenever $((x_{j})_{j\in J}, x)\in {\mathcal {S}}$ and $x\in U$, then eventually $x_{j}\in U\}$ is a topology.

### *Remark 2*

For dcpos the preceding definition of ${\mathcal {S}}$-limit is equivalent to the standard one (Gierz et al. 2003, Definition II-1.1) (as in a dcpo, $x\in D^{\delta }$ means $x\in \downarrow \vee D$).

### **Lemma 5**

*LetPbe a poset*, *then*$\mathcal {O({\mathcal {S}})}=\sigma _2(P)$.

### *Proof*

First, suppose that $U\in \mathcal {O({\mathcal {S}})}$. To prove $U=\uparrow U$, assume that $u\in U$ and $u\le x$. Then $u\le x\equiv _{S}$ lim *x* with the constant net (*x*) with value *x*. So by the definition $((x),u)\in {\mathcal {S}}$. Since we have $u\in U\in \mathcal {O({\mathcal {S}})}$, we conclude from the definition of $\mathcal {O({\mathcal {S}})}$ that the net (*x*) must be eventually in *U*. This means $x\in U$. In order to show that $D^{\delta }\cap U\ne \emptyset \Rightarrow U\cap D\ne \emptyset$ for each directed set $D\subseteq P$, let $x\in D^{\delta }\cap U\ne \emptyset$ . Consider the net $(x_{d})_{d\in D}$ with $x_{d}=d$. Now since $((x_{d})_{d\in D}, x)\in {\mathcal {S}}$, we conclude that $d=x_{d}$ is eventually in *U*; whence $D\cap U\ne \emptyset$.

Conversely, suppose that $U\in \sigma _{2}(P)$. For any $((x_{j})_{j\in J}, x)\in {\mathcal {S}}$ with $x\in U$, by the definition of ${\mathcal {S}}$,we have $x\in D^{\delta }$ for some directed set *D* of eventual lower bounds of the net $(x_{j})_{j\in J}$. Now $x\in D^{\delta }\cap U$, and then $u\in D$ for some $u\in U$ by the definition of $\sigma _{2}(P)$. By definition $u\le x_{j}$ for all $k\le j$ for some $k\in J$. By $U=\uparrow U$, $x_{j}\in U$ holds eventually. Hence $U\in \mathcal {O({\mathcal {S}})}$.$\square$

### **Lemma 6**

*LetPbe an*$s_2$-*continuous poset*. *Then for any*$x\in P$, $\Uparrow x\in \sigma _{2}(P)$.

### *Proof*

It follows from Lemma 1.$\square$

### **Lemma 7**

*LetPbe a poset and*$y\in int_{\sigma _{2}(P)}\uparrow x$. *Then*$x\ll y$, *where*$int_{\sigma _{2}(P)}\uparrow x$*denotes the interior of*$\uparrow x$*with respect to the weak Scott topology*$\sigma _{2}(P)$.

### *Proof*

Let $y\in int_{\sigma _{2}(P)}\uparrow x$. For any directed set *D* with $y\in D^{\delta }$, we have $D^{\delta }\cap int_{\sigma _{2}(P)}\uparrow x\ne \emptyset$, and whence $int_{\sigma _{2}(P)}\uparrow x\cap D\ne \emptyset$. Thus there is $d\in int_{\sigma _{2}(P)}\uparrow x\cap D$. Now we have $x\le d$ and $d\in D$. Therefore $x\ll y$.$\square$

### **Proposition 1**

*LetPbe an*$$s_2$$-*continuous poset. Then*$$x\equiv _{{\mathcal {S}}}lim$$$$x_{j}$$*if and only if the net*$$(x_{j})_{j\in J}$$*converges to the elementxwith respect to the weak Scott topology*$$\sigma _{2}(P)$$. *That is, the*$${\mathcal {S}}$$-*convergence is topological*.

### *Proof*

The necessity follows from Lemma 5. Now suppose that the net $(x_{j})_{j\in J}$ converges to an element *x* with respect to the weak Scott topology. For all $y\in \Downarrow x$, we have $x\in \Uparrow y\in \sigma _{2}(P)$ by Lemma 6. Thus there is $k\in J$ such that $x_{j}\in \Uparrow y$ for all $j\ge k$. Since *P* is $s_2$-continuous, $x\in (\Downarrow x)^{\delta }$ and $\Downarrow x$ is directed. Hence we have $((x_{j})_{j\in J}, x)\in {\mathcal {S}}$, that is, $x\equiv _{\mathcal {S}}$lim $x_{j}$.$\square$

### **Proposition 2**

*LetPbe a poset*. *If the*${\mathcal {S}}$-*convergence is topological, thenPis*$s_2$-*continuous*.

### *Proof*

By Lemma 5, the topology induced by ${\mathcal {S}}$-convergence is the weak Scott topology. So if the ${\mathcal {S}}$-convergence is topological, then we must have $x\equiv _{\mathcal {S}}$lim $x_{j}$ if and only if the net $(x_{j})_{j\in J}$ converges to the element *x* in the weak Scott topology. For any $x\in P$, let $J=\{(U, n, a)\in N(x)\times {\mathbb {N}}\times P: a\in U\}$, where *N*(*x*) consists of all weak Scott open sets containing *x*, and define an order on *J* to be the lexicographic order on the first two coordinates, i.e., $(U, m, a)\le (V, n, b)$ if and only if *V* is proper subset of *U* or $U=V$ and $m\le n$. Put $x_{j}=a$ for each $j=(U, m, a)\in J$. Then it is not difficult to check that the net $(x_{j})_{j\in J}$ converges to *x* with respect to the weak Scott topology, and hence $x\equiv _{\mathcal {S}}$lim $x_{j}$. Thus there is a directed set *D* of eventual lower bounds of the net $(x_{j})_{j\in J}$ such that $x\in D^{\delta }$. If $d\in D$, then there is $k=(U, m, a)\in J$ such that $(V, n, b)=j\ge k$ implies $d\le x_{j}=b$. Specially we have $(U, m+1, b)\ge (U, m, a)=k$ for all $b\in U$. Therefore $x\in U\subseteq \uparrow d$. It follows that $D\subseteq \downarrow x$ and $x\in int_{\sigma _{2}(P)}\uparrow d$. By Lemma 7$d\ll x$, and then $D\subseteq \Downarrow x$. Thus $x\in D^{\delta }\subseteq (\Downarrow x)^{\delta }$. Obviously, $\Downarrow x$ is directed. Hence *P* is $s_2$-continuous.$\square$

From Propositions 1 and 2, we immediately have:

### **Theorem 2**

*LetPbe a poset. Then the following conditions are equivalent:*

1. *Pis*$$s_2$$-*continuous*;
2. *The*${\mathcal {S}}$-*convergence inPis topological for the weak Scott topology, that is, for all*$x\in P$*and all nets*$(x_{j})_{j\in J}$*inP*, $x\equiv _{\mathcal {S}}$lim $x_{j}$*if and only if*$(x_{j})_{j\in J}$*converges to the elementxwith respect to the weak Scott topology*.

### **Corollary 1**

(Gierz et al. 2003) *LetPbe a dcpo*. *Then the following conditions are equivalent*:

1. *Pis a domain*;
2. *The*$${\mathcal {S}}$$-*convergence inPis topological for the Scott topology, that is, for all*$$x\in P$$*and all nets*$$(x_{j})_{j\in J}$$*inP*, $$x\equiv _{\mathcal {S}}lim$$$$x_{j}$$*if and only if*$$(x_{j})_{j\in J}$$*converges to the elementxwith respect to the Scott topology*.

## ${\mathcal {GS}}$-Convergence in $s_2$-quasicontinuous posets 

In this section, the concept of ${\mathcal {GS}}$-convergence in a poset is introduced. It is proved that the poset *P* is $s_2$-quasicontinuous if and only if the ${\mathcal {GS}}$-convergence in *P* is topological.

### **Definition 6**

Let *P* be a poset and $(x_{j})_{j\in J}$ a net in *P*. $F\subseteq P$ is called a quasi-eventual lower bound of a net $(x_{j})_{j\in J}$ in *P*, if *F* is finite and there exists $k\in J$ such that $x_{j}\in \uparrow F$ for all $j\ge k$.

Obviously, an eventual lower bound is the quasi-eventual lower bound.

### **Definition 7**

Let *P* be a poset and $(x_{j})_{j\in J}$ a net in *P*. *x* is called a ${\mathcal {GS}}$-limit of the net $(x_{j})_{j\in J}$ if there exists a directed family ${\mathcal {F}}=\{F\subseteq P: F$ is finite} of quasi-eventual lower bounds of the net $(x_{j})_{j\in J}$ in *P* such that $\bigcap _{F\in {\mathcal {F}}}\uparrow F\subseteq \uparrow x$. We also say $(x_{j})_{j\in J}$ quasi ${\mathcal {S}}$ converges to *x* and write $x\equiv _{{\mathcal {GS}}}$lim $x_{j}$.

### **Lemma 8**

*An*${\mathcal {S}}$-*limit of the net*$(x_{j})_{j\in J}$*must be a*${\mathcal {GS}}$-*limit of the net*$(x_{j})_{j\in J}$.

### *Proof*

Let *P* be a poset and $(x_{j})_{j\in J}$ a net with $x\equiv _{\mathcal {S}}$lim $x_{j}$. Then there is a directed set *D* of eventual lower bounds of the net $(x_{j})_{j\in J}$ with $x\in D^{\delta }$. Let ${\mathcal {F}}=\{\{d\}: d\in D\}$, then ${\mathcal {F}}$ is a directed family of quasi-eventual lower bounds of the net $(x_{j})_{j\in J}$ and $D^{\uparrow }=\bigcap \{\uparrow d: d\in D\}\subseteq \uparrow x$. Thus $x\equiv _{{\mathcal {GS}}}$lim $x_{j}$.$\square$

### *Remark 3*

A ${\mathcal {GS}}$-limit of the net $(x_{j})_{j\in J}$ may not be an ${\mathcal {S}}$-limit of the net $(x_{j})_{j\in J}$.

### *Example 4*

Let $P={\mathbf {N}}\cup \{\top , z\}$, where ${\mathbf {N}}$ denotes the set of all natural numbers. The order $\le$ on *P* is defined as follows:

1. $$\forall x\in P, x\le \top $$;
2. $\forall x, y\in {\mathbf {N}}, x\le y$ if *x* is less than or equal to *y* according to the usual order on natural numbers.

Then *P* is $s_2$-quasicontinuous but not $s_2$-continuous. Also for all $n\in {\mathbf {N}}, \{z, n\}\ll z$ and $\uparrow z=\bigcap _{n\in {\mathbf {N}}}\uparrow \{z, n\}$. Let $x_{2n}=n, x_{2n+1}=z$, then $(x_{j})_{j\in {\mathbf {N}}}$ is a net and $\{z, n\}$ is a quasi-eventual lower bound of it. Hence $z\equiv _{{\mathcal {GS}}}$lim $x_{n}$. It is not difficult to check that $z\le x_{n}$ does not hold eventually. Thus *z* is not an ${\mathcal {S}}$-limit of the net $(x_{n})_{n\in {\mathbf {N}}}$.

### **Proposition 3**

*Let*${\mathcal {F}}$*be a directed family of nonempty finite sets in a posetP*. *If*$x\in U\in \sigma _2(P)$*and*$\bigcap _{F\in {\mathcal {F}}}\uparrow F\subseteq \uparrow x$, *then*$F\subseteq U$*for some*$F\in {\mathcal {F}}$.

### *Proof*

Suppose not, then the collection $\{F\backslash U: F\in {\mathcal {F}}\}$ is a directed family of nonempty finite sets. By Lemma 4, there is some directed set $D\subseteq \bigcup \{F\backslash U: F\in {\mathcal {F}}\}$ such that $D\cap (F\backslash U)\ne \emptyset$ for all $F\in {\mathcal {F}}$. Then $D^{\uparrow }=\bigcap _{d\in D}\uparrow d\subseteq \bigcap _{F\in {\mathcal {F}}}\uparrow (F\backslash U)\subseteq \bigcap _{F\in {\mathcal {F}}}\uparrow F\subseteq \uparrow x$. Thus $x\in (D^{\uparrow })^{\downarrow }=D^{\delta }$. Now we have $x\in D^{\delta }\cap U\ne \emptyset$, and hence $D\cap U\ne \emptyset$ by the definition of the weak Scott open set, that is, there is some $d\in D$ with $d\in U$. But this contradicts $d\in F\setminus U$ for some $F\in {\mathcal {F}}$.$\square$

Let ${\mathcal {GS}}$ denote the class of those pairs $((x_{j})_{j\in J}, x)$ with $x\equiv _{{\mathcal {GS}}}$lim $x_{j}$, then $\mathcal {O({\mathcal {GS}})}=\{U\subseteq P:$ whenever $((x_{j})_{j\in J}, x)\in {\mathcal {GS}}$ and $x\in U$, then eventually $x_{j}\in U\}$ is also a topology.

Though ${\mathcal {S}}$-limit and ${\mathcal {GS}}$-limit of the net $(x_{j})_{j\in J}$ are different, they may generate the same topology.

### **Proposition 4**

*LetPbe a poset, then*$\mathcal {O({\mathcal {GS}})}=\mathcal {O(S)}=\sigma _2(P)$.

### *Proof*

By Lemma 5, we only need to show that $\mathcal {O({\mathcal {GS}})}=\sigma _2(P)$. By Lemma 8, we have ${\mathcal {S}}\subseteq {\mathcal {GS}}$, so $\mathcal {O({\mathcal {GS}})}\subseteq \sigma _2(P)$. Conversely, let $U\in \sigma _2(P)$ and $((x_{j})_{j\in J}, x)\in {\mathcal {GS}}$ with $x\in U$. Since $x\equiv _{{\mathcal {GS}}}$lim $x_{j}$, there is a directed family ${\mathcal {F}}=\{F\subseteq P: F$ is finite} of quasi-eventual lower bounds of a net $(x_{j})_{j\in J}$ in *P* such that $\bigcap _{F\in {\mathcal {F}}}\uparrow F\subseteq \uparrow x$. By Proposition 3 there is $F\in {\mathcal {F}}$ such that $\uparrow F\subseteq U$. Notice that *F* is a quasi-eventual lower bound of a net $(x_{j})_{j\in J}$, there is some $j_{0}\in J$ such that $x_{j}\in \uparrow F\subseteq U$ for all $j\ge j_{0}$. Thus $U\in \mathcal {O({\mathcal {GS}})}$.$\square$

Now we derive the ${\mathcal {GS}}$-convergence in the $s_2$-quasicontinuous poset is topological.

### **Proposition 5**

*LetPbe an*$$s_2$$-*quasicontinuous poset*. *Then*$$x\equiv _{{\mathcal {GS}}}lim$$$$x_{j}$$*if and only if the net*$$(x_{j})_{j\in J}$$*converges to the elementxwith respect to the weak Scott topology*.

### *Proof*

The necessity follows from Proposition 4. Now suppose that the net $(x_{j})_{j\in J}$ converges to an element *x* with respect to the weak Scott topology. Since *P* is $s_2$-quasicontinuous, there exists a directed family $w(x)=\{F\subseteq P:F\in P^{(<\omega )}$ and $F\ll x\}$ and $\uparrow x=\bigcap \{\uparrow F:F\in w(x)$ }. For all $F\in w(x)$, let $U_{F}=\{y\in P: F\ll y\}$. Then $U_{F}\in \sigma _{2}(P)$ and $x\in U_{F}$ by Lemma 3, and hence $x_{j}\in U_{F}$ eventually holds. Thus *F* is a quasi-eventual lower bound of the net $(x_{j})_{j\in J}$ and $x\equiv _{{\mathcal {GS}}}$lim $x_{j}$.$\square$

The converse is also true.

### **Proposition 6**

*LetPbe a poset*. *If the*${\mathcal {GS}}$-*convergence is topological*, *thenPis*$s_2$-*quasicontinuous*.

### *Proof*

Suppose that the ${\mathcal {GS}}$-convergence is topological. Then $x\equiv _{{\mathcal {GS}}}$lim $x_{j}$ if and only if the net $(x_{j})_{j\in J}$ converges to the element *x* with respect to the weak Scott topology $\sigma _{2}(P)$ by Proposition 4.

For any $x\in P$, let $J=\{(U, n, a)\in N(x)\times {\mathbf {N}}\times P: a\in U\}$, where *N*(*x*) consists of all weak Scott open sets containing *x*, and define an order on *J* to be the lexicographic order on the first two coordinates. That is, $(U, m, a)\le (V, n, b)$ if and only if *V* is proper subset of *U* or $U=V$ and $m\le n$. Obviously, *J* is directed. Let $x_{j}=a$ for all $j=(U, m, a)\in J$. Then it is not difficult to check that the net $(x_{j})_{j\in J}$ converges to the element *x* with respect to the weak Scott topology, and hence $x\equiv _{{\mathcal {GS}}}$lim $x_{j}$. Thus it concludes that there is a directed family ${\mathcal {F}}=\{ F\subseteq P: F$ is finite} of quasi-eventual lower bounds of the net $(x_{j})_{j\in J}$ in *P* such that $\bigcap _{F\in {\mathcal {F}}}\uparrow F\subseteq \uparrow x$. Now we prove that (1) for all $F\in {\mathcal {F}}, F\ll x$; $(2) \bigcap _{F\in {\mathcal {F}}}\uparrow F= \uparrow x$.

1. Let $D\subseteq P$ be directed with $x\in D^{\delta }$. Since *F* is a quasi-eventual lower bound of the net $(x_{j})_{j\in J}$, there is $j_{0}=(U, m, a)\in J$ such that $x_{j}\in \uparrow F$ for all $j=(V, n, b)>j_{0}$. Notice $x\in U$, so $D\cap U\ne \emptyset$. Pick $d\in D\cap U$. Set $i=(U, m+1, d)$, then $i>(U, m, a)=j_{0}$. Thus $d=x_{i}\in \uparrow F$, that is, $F\ll x$.
2. We only need to show that $\uparrow x\subseteq \bigcap _{F\in {\mathcal {F}}}\uparrow F$. Suppose not, then there exists $y\ge x$ but $y\notin \bigcap _{F\in {\mathcal {F}}}\uparrow F$, that is, there exists $F\in {\mathcal {F}}$ with $y\notin \uparrow F$. And then $\uparrow F\subseteq P\backslash \downarrow x$. Again since *F* is a quasi-eventual lower bound of the net $(x_{j})_{j\in J}$, there exists $j_{0}=(U, m, a)\in J$ such that $x_{j}\in \uparrow F$ for all $j=(V, n, b)>j_{0}$. Now we have $x\in U$. Set $i=(U, m+1, x)$, then $i>(U, m, x)=j_{0}$. Thus $x=x_{i}\in \uparrow F\subseteq P\backslash \downarrow x$, a contradiction.

$$\square $$

From Propositions 5 and 6 we have:

### **Theorem 3**

*LetPbe a poset*. *Then the following conditions are equivalent*:

1. *Pis*$$s_2$$-*quasicontinuous*;
2. *The*$${\mathcal {GS}}$$-*convergence inPis topological for the weak Scott topology*$$\sigma _2(P)$$, *that is, for all*$$x\in P$$*and all nets*$$(x_{j})_{j\in J}$$*inP*, $$x\equiv _{{\mathcal {GS}}}lim$$$$x_{j}$$*if and only if*$$(x_{j})_{j\in J}$$*converges toxwith respect to the weak Scott topology*.

### **Corollary 2**

(Zhou and Li 2013) *LetPbe a dcpo*. *Then the following conditions are equivalent*:

1. *Pis a quasicontinuous domain*;
2. $$S^{*}$$-*convergence inPis topological for the Scott topology*$$\sigma (P)$$, *that is, for all*$$x\in P$$*and all nets*$$(x_{j})_{j\in J}$$*inP*, $$(x_{j})_{j\in J}$$$$S^{*}$$*converges toxif and only if*$$(x_{j})_{j\in J}$$*converges toxwith respect to the Scott topology*.

## Conclusions

In this paper, we present one way to generalize ${\mathcal {S}}$-convergence and ${\mathcal {GS}}$-convergence of nets for arbitrary posets by use of the cut operator instead of joins and come to the main conclusions are: (1) A poset *P* is $s_2$-continuous if and only if the ${\mathcal {S}}$-convergence in *P* is topological; (2) *P* is $s_2$-quasicontinuous if and only if the ${\mathcal {GS}}$-convergence in *P* is topological.
